# Pathway-dependent cold activation of heat-responsive TRPV channels

**DOI:** 10.21203/rs.3.rs-6450204/v1

**Published:** 2025-04-16

**Authors:** Guangyu Wang

**Affiliations:** Department of Physiology and Membrane Biology, University of California School of Medicine, Davis, CA, USA

**Keywords:** noncovalent thermoring structure, protein unfolding threshold, symmetric thermosensitivity

## Abstract

The homotetrameric thermosensitive transient receptor potential vanilloid 1–4 (TRPV1-4) channels in sensory neurons are highly responsive to heat stimuli. However, their primary heat sensors or triggers for heat activation have not been examined for cold activation. In this study, cold activation of minimal TRPV1 without the pore turret was compared with that of full-length human TRPV3. The former followed a pathway from the putative heat activation starter, while the latter tracked a different pathway starting far from the assumed heat activation point. The results showed that the former shared temperature sensitivity with heat activation while the latter did not. Therefore, this mirrored thermosensitivity can be used to confirm the location of the primary thermal sensor for TRPV1 or TRPV3, and potentially define the primary thermal sensor of other thermosensitive proteins like TRPV2 or TRPV4 once the same heat capacity mechanism is applied.

## Introduction

Thermosensitive transient receptor potential (TRP) channels, such as TRP vanilloid 1–4 (TRPV1-4), TRP melastatin 2–5, 8 (TRPM2-5, 8), TRP ankyrin 1 (TRPA1) and TRP canonical 5 (TRPC5), function as biological thermometers. They have a high temperature coefficient or sensitivity (Q_10,_ the rate of activity increase of TRP channels over a 10°C rise) and specific activation thresholds within a temperature range of 17°C to 52°C (1–16). While various regions have been proposed as the primary thermal sensor or trigger for the specific threshold, such as the ankyrin repeat domain (ARD), pre-S1 domain, S1-S4 domain, pore domain, TRP helix and C terminal domain (CTD) (17–38), recent temperature-dependent cryogenic electron microscopy (cryo-EM) structures of native TRPV1, TRPV3 and TRPM8 channels have shown a global cooperative conformational change across all these regions during channel opening (39–42). Furthermore, recent data from differential scanning calorimetry (DSC) and electrophysiological recordings have indicated the same specific temperature thresholds for both channel opening and melting in either TRPV1 or TRPV2 (43–44). Therefore, the heat-induced unfolding of a particular noncovalent interaction is essential to initiate channel opening.

Recently, a graph theory-based grid thermodynamic model has been developed to identify the weakest noncovalent interaction responsible for the melting threshold of a protein (45–51). In this model, noncovalent interactions identified in the high-resolution 3D structure of the protein can form an adjacency matrix or a systematic fluidic grid-like mesh network along the single polypeptide chain. In this network, each node represents a protein residue and two nodes are linked as an edge if a pair of protein residues form a noncovalent interaction. For each noncovalent interaction, the shortest round path between two nodes along the single polypeptide chain and other edges can generate a constrained topological grid to control the melting temperature threshold (T_m,th_) of that noncovalent interaction. In this regard, the constrained grid can be defined as a thermo-sensitive ring or a thermo-ring with the path length being its size. Thus, the T_m,th_ for heat unfolding of a given protein can be determined by the grid size and the strength of the least-stable noncovalent interaction in the biggest grid; In addition, the systematic thermal instability (T_i_) or flexibility can be computed as the ratio of the total unshared grid sizes (S) to the total noncovalent interactions (N) along the same single polypeptide chain. Finally, the structural temperature sensitivity (Ω_10_) can be defined as a change in the total chemical potentials of grids upon a change in the total noncovalent interactions along the same gating pathway. Since three parameters are consistent with the experimental values, the weakest noncovalent link along the lipid-dependent minimal gating pathway may serve as the primary thermal sensor or starter for channel activation of TRPV1, TRPV3 or TRPM8 (48–50). However, TRP channels are homo-tetramers and the inter-subunit interaction may also act as a primary thermal sensor or starter to initiate channel activation. Given that the heat and cold unfolding of any protein from the same starter has the same or comparable temperature coefficient (52), this principle was used to examine the primary thermal sensor or starter of thermosensitive TRPV channels.

In this study, the intra-subunit active vanilloid site of the minimal thermosensitive rat TRPV1 (rTRPV1) channel without the pore turret (604–626) was utilized as a positive control. The purpose was to investigate whether the removal of phosphatidylinositol (PI) from this site disrupts the nearby weakest noncovalent interaction along the PI-dependent minimal gating pathway, initiating both heat and cold activation with a shared temperature coefficient (53–54). On the other hand, the highly conserved cytoplasmic ATP site shared among TRPV1, TRPV2 and TRPV3 was used as a negative control. The goal was to determine whether disrupting the highly conserved inter-subunit K169-E751’ salt bridge at the interface between the ARD and the CTD’ in reduced human TRPV3 (hTRPV3) through the K169A mutation triggers cold activation, which has an unshared temperature coefficient compared to the heat activation induced by breaking the weakest noncovalent interaction in the pore domain along the phosphatidylcholine (PC)-dependent minimal gating pathway (55–57). The results demonstrated that the primary thermal sensor or starter actually originates from the weakest intra-subunit noncovalent interaction along the single polypeptide chain rather than any other inter-subunit noncovalent interactions.

## Results

### Reduced closed rTRPV1-Δ(604–826) in MSP2N2 has a corresponding melting threshold to release PI from the vanilloid site for heat activation

**Figure 1:**
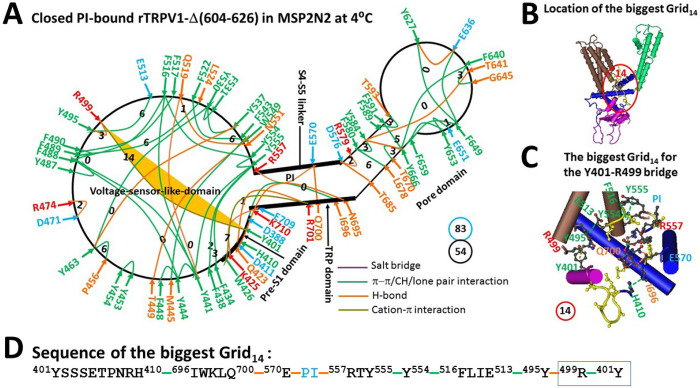
The grid-like noncovalently interacting mesh network along the PI-dependent minimal gating pathway of the reduced rTRPV1-D(604-626) channel in a closed state at 4 °C. (**A)** The topological grid network is depicted using the cryo-EM structure of a single subunit of the reduced and closed rTRPV1-D(604-626) channel in MSP2N2 at 4 °C (PDB ID, 5IRZ). The pore domain, the S4-S5 linker, the TRP domain, and the pre-S1 domain are indicated by black arrows, except the VSLD. Salt bridges, p interactions, and H-bonds between paired amino acid side chains along the PI-dependent minimal gating pathway from D388 to K710 are denoted in purple, green, and orange, respectively. The specific grid sizes necessary to regulate the least-stable noncovalent interactions in the grids are indicated with black numbers. The weakest Y401-R499 cation-p interaction in the biggest Grid_14_ is emphasized in yellow. The total grid sizes and grid size-controlled noncovalent interactions along the PI-dependent minimal gating pathway are displayed in cyan and black circles, respectively. (**B)** The position of the biggest Grid_14_. (**C)** The structure of the biggest Grid_14_ with a 14-residue size to regulate the weakest Y401-R499 cation-p interaction at the pre-S1/VSLD interface. (**D)** The sequence of the biggest Grid_14_ to control the weakest Y401-R499 cation-p interaction highlighted in the blue box.

When the pore turret (604–626) is removed from rTRPV1, this minimal thermosensitive channel is structured from N-terminal T335 to C-terminal T751 in MSP2N2 at 4°C (5IRZ) (53). When compared with the full-length rTRPV1 in MSP2N2 at 48°C (7LPC), most of the noncovalent interactions were conserved except for small fractional changes along the PI-dependent minimal gating pathway from D388 in the pre-S1 domain to K710 in the TRP domain (Fig. 1A). In the pore domain, the E636-K639 H-bond was disrupted and the π interaction moved from D654-F655 to E651-Y653. At the interface between the S4-S5 linker and the TRP domain, the E570-K571-E692-R575 salt bridges and the Q691-N695 H-bond were disconnected, and the E570 H-bonded with Q700 via their side chains. At the interface between the S4-S5 linker and the voltage sensor-like domain (VSLD), the D509-PI-R557 bridge was broken. At the interface between the TRP domain and the pre-S1 domain, the Q700-PI-R409 bridge was also disrupted together with the D707-Y401 and K710-E397 H-bonds. At the interface between the VSLD and the pre-S1 domain, the R409-PI-D509 bridge and the W426-F434 π interactions were also broken. In the VSLD, the R534-E536 salt bridge was disconnected along with the shifts of the H-bond from Y487-R491 to Y495-R499 and the π interaction from F517-L521 to F489-F517. Finally, the Y441-Q519 H-bond appeared. Therefore, when the total noncovalent interactions and grid sizes decreased from 63 and 87 to 54 and 83, respectively (Fig. 1A), the systematic thermal instability (T_i_) increased from 1.38 to 1.54 (Table 1) (48).

After disrupting the inhibitory Y401-D707 and E397-K710 H-bonds (48), the biggest Grid_14_ was found to control the same least-stable Y401-R499 cation-π interaction but through a different thermoring pathway. It cycled through Y401 to H410, I696, Q700, E570, PI, R557, Y555, Y554, F516, E513, Y495, R499, and back to Y401 (Fig. 1B–D). Therefore, the weakest Y401-R499 bridge was closely related to PI binding. When this bridge was energetically equivalent to 1.5 basic H-bonds (1.5 kcal/mol), the calculated melting temperature threshold (T_m,th_) was approximately 41°C, matching the experimental value of 41°C for heat activation (58). Accordingly, in line with previous research, the heat-induced unfolding of the weakest Y401-R499 bridge was essential to release PI from the active vanilloid site, allowing channel opening above 41°C (40, 48). The next question is whether the removal of PI also disrupts the same weakest Y401-R499 bridge to faciliate channel opening at lower temperatures with a shared thermosensitivity.

#### Removal of PI from reduced rTRPV1-Δ(604–826) in MSP2N2 induces cold activation with a shared thermosensitivity

**Figure 2:**
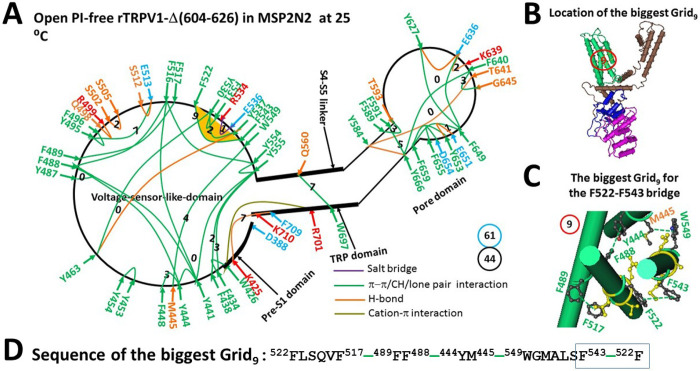
The grid-like noncovalently interacting mesh network along the PI-dependent minimal gating pathway of the reduced rTRPV1-D(604-626) channel in an open state at 25 °C. (**A)** The topological grid network is depicted using the cryo-EM structure of a single subunit of the reduced and open rTRPV1-D(604-626) channel in MSP2N2 at 25 °C (PDB ID, 8U3L). The pore domain, the S4-S5 linker, the TRP domain, and the pre-S1 domain are indicated by black arrows, except the VSLD. Salt bridges, p interactions, and H-bonds between paired amino acid side chains along the PI-dependent minimal gating pathway from D388 to K710 are denoted in purple, green, and orange, respectively. The specific grid sizes necessary to regulate the least-stable noncovalent interactions in the grids are indicated with black numbers. The weakest F522-F543 p-p interaction in the biggest Grid_9_ is emphasized in yellow. The total grid sizes and grid size-controlled noncovalent interactions along the PI-dependent minimal gating pathway are displayed in cyan and black circles, respectively. (**B)** The position of the biggest Grid_9_. (**C)** The structure of the biggest Grid_9_ with a 9-residue size to regulate the weakest F522-F543 p-p interaction in the VSLD. (**D)** The sequence of the biggest Grid_9_ to control the weakest F522-F543 p-p interaction highlighted in the blue box.

When the PI lipid was removed from the active vanilloid site to disrupt the weakest Y401-R499 cation-π interaction and the R557-PI-E570 bridge along the PI-dependent minimal gating pathway from D388 to K710, rTRPV1-Δ(604–826) was open in MSP2N2 at 25°C with a global cooperative conformational change (Fig. 2A). As a result, the H410-I696 π interaction and the D411-N695 and Q423-R701 H-bonds were broken at the pre-S1/TRP interface, and the E570-Q700 H-bond was replaced with the Q560-W697 π interaction at the S4-S5 linker/TRP interface. This conformational change also disrupted the R579-D576-T685 H-bonds and the Y584-F580-L678 and T670-Y666 π interactions but induced the formation of the E636-K639 H-bond and the D654-F655 π interaction. In the VSLD, in addition to disrupting the Y441-Q519-N551 and W549-T449 H-bonds and the F489-L524 π interaction, some small changes were observed, such as the disappearance of the P456-Y463 and F489-F490 π interactions and the D471-R474 and Y495-R499 H-bonds. However, the W425-F434 and Y495-F496 π interactions and the Q498-R499 and S502-S505 and S512-E513 H-bonds, along with the R534-E536 salt bridge, appeared.

Overall, the totals of noncovalent interactions and grid sizes decreased from 54 and 83 to 44 and 61, respectively (Figs. 1A & 2A). Consequently, the systematic thermal instability (T_i_) decreased from 1.54 to 1.39 (Table 1). Of particular interest, along with the disassociation of the same weakest Y401-R499 bridge at the pre-S1/VSLD interface, the calculated cold thermosensitivity (Ω_10,cold_) was 19.1, which was comparable to the measured heat thermosensitivity (Q_10,heat_) of 18.5 (58).

On the other hand, the new biggest Grid_9_ was introduced to control the least-stable F522-F543 π interaction in the VSLD through a thermoring from F522 to F517, F489, Y444, M445, W549, F543, and back to F522 (Fig. 2B–D). This bridge was highly conserved in gating states of rTRPV1, regardless of the deletion of the pore turret from 604 to 626 (48). Therefore, it may play an important role in stabilizing the rTRPV1 channel. When this weakest bridge energetically equated to 0.5 basic H-bond (0.5 kcal/mol), the calculated melting threshold (T_m,th_) was approximately 41°C (Table 1). Since no grid size exceeded 9 between the C- and N-termini beyond the PI-dependent minimal gating pathway from D388 to K710 (Figure S1), this open state induced by PI removal at low temperatures may only occur below 41°C.

#### Reduced closed hTRPV3 in MSP2N2 has a matching melting threshold for the initial heat activation

**Figure 3:**
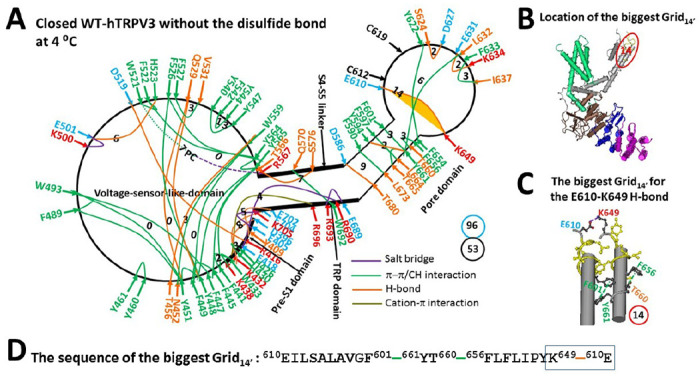
The grid-like noncovalently interacting mesh network along the PC-dependent minimal gating pathway of the reduced hTRPV3 channel in a closed state at 4 °C. (**A)** The topological grid network is illustrated using the cryo-EM structure of a single subunit of the reduced and closed hTRPV3 channel in MSP2N2 at 4 °C (PDB ID, 6UW4). The pore domain, the S4-S5 linker, the TRP domain, and the pre-S1 domain are indicated by black arrows, except the VSLD. Salt bridges, p interactions, and H-bonds between paired amino acid side chains along the PC-dependent minimal gating pathway from D396 to K705 are denoted in purple, green, and orange, respectively. The specific grid sizes necessary to regulate the least-stable noncovalent interactions in the grids are indicated with black numbers. The weakest E610-K649 H-bond in the biggest Grid_14′_ is emphasized in yellow. The dashed line represents the putative PC bridge between W521 and R567. The total grid sizes and the total grid size-controlled noncovalent interactions along the PC-dependent minimal gating pathway are displayed in cyan and black circles, respectively. (**B)** The position of the biggest Grid_14′_. (**C)** The structure of the biggest Grid_14′_ with a 14-residue size to regulate the wesakest E610-K649 H-bond in the pore domain. (**D)** The sequence of the biggest Grid_14′_ to control the weakest E610-K649 H-bond highlighted in the blue box.

Reduced hTRPV3 showed structural similarities to reduced mTRPV3 at 4°C, including the active vanilloid site for the same phosphatidylcholine (PC) lipid binding through a π interaction with W521 and a salt bridge with R567 (Fig. 3A) (39, 49, 57). However, some differences were observed throughout the entire polypeptide chain. At the pre-S1/VSLD interface, the T411-D519 H-bond was disrupted. along with the formation of the H426-H430 π–π interaction. When the D519-R698 salt bridge at the VSLD/TRP interface was replaced with the E418-R690 salt bridge at the pre-S1/TRP interface, the H-bond moved from T397-E704 to Y409-E702 together with the formation of the E405-K705 salt bridge. Meanwhile, the Q570-E689 H-bond was also substituted by the Q570-W692 π interaction. As a result, in the VSLD, the T456-W559 H-bond appeared with the Y451-F489 π interaction, the Q514-S518 H-bond was replaced with the K500-E501 salt bridge and the E501-H523 H-bond, and the W521-F524-V528 π interactions were substituted by the F542-Y544 π interaction. In the pore domain, the H-bond moved from K614-N647 to E610-K649, along with the replacement of the F625-V629 and T649-Y650 π interactions and the Y594-T636-Y661 H-bonds with the S624-D627 and E631-K634 H-bonds. Collectively, the total noncovalent interactions along the PC-dependent minimal gating pathway from D396 in the pre-S1 domain to K705 in the TRP domain increased from 51 to 53 despite the same total grid sizes of 96 (Fig. 3A). Consequently, the systematic thermal instability (T_i_) decreased from 1.88 to 1.81 (Table 1)(49).

Notably, the R416-D519 H-bond at the pre-S1/VSLD interface was not controlled by the smaller thermoring (Grid_4_) from R416 to T411, D519 and back to R416 (49). Instead, it was governed by the smaller thermoring (Grid_3_) from R416 to E418, R690, E689, R693, W692, Q570, T566, Y565, Y564, F522, W521, D519 and back to R416 (Fig. 3A). Since no grid size was bigger than 14 between the C- and N-termini beyond the PC-dependent minimal gating pathway from D396 to K705 (Figure S1), Grid_14_ in the pore domain was still the biggest (Fig. 3A). It had the shortest circular path from E610 to F601, Y661, T660, F656, K649, and back to E610 to control the least-stable E610-K649 H-bond/salt bridge between two pore turrets (Fig. 3B–D). With 2.5 equivalent basic H-bonds being energetically equivalent to this weakest bridge, the calculated T_m,th_ for heat unfolding was at least 51°C (Table 1), which was close to the measured threshold of 50°C for the heat-induced channel opening of the reduced hTRPV3 channel (32).

### Disrupting the inter-subunit K169-E751’ salt bridge in reduced hTRPV3 in MSP2N2 induces cold activation with unique thermosensitivity

If heat-evoked hTRPV3 activation starts with the disruption of the intersubunit K169-E751’ bridge rather than the weakest intra-subunit E610-K649 bridge, then the same thermosensitivity for heat activation should be observed for cold activation. To test this hypothesis, the thermoring of the K169A-induced open state at 4°C was analyzed.

**Figure 4:**
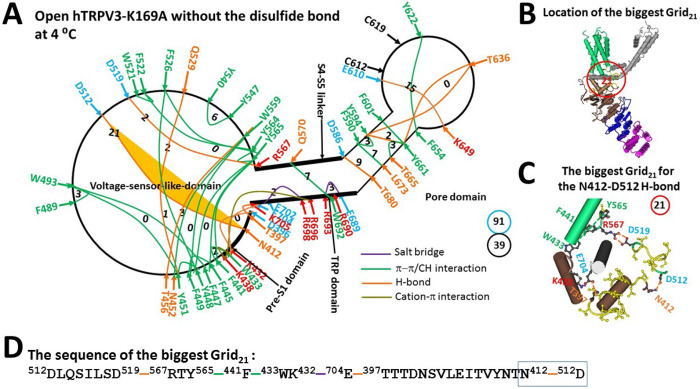
The grid-like noncovalently interacting mesh network along the PC-dependent minimal gating pathway of the reduced hTRPV3-K169A channel in an open state at 4 °C. (**A)** The topological grid network is shown using the cryo-EM structure of a single subunit of the reduced and open hTRPV3-K169A channel in MSP2N2 at 4 °C (PDB ID, 6UW6). The pore domain, the S4-S5 linker, the TRP domain, and the pre-S1 domain are indicated by black arrows except the VSLD. Salt bridges, p interactions, and H-bonds between paired amino acid side chains along the PC-dependent minimal gating pathway from D396 to K705 are marked in purple, green, and orange, respectively. The specific grid sizes needed to control the least-stable noncovalent interactions in the grids are labeled with black numbers. The weakest N412-D512 H-bond in the biggest Grid_21_ is highlighted. The total grid sizes and the total grid size-controlled noncovalent interactions along the PC-dependent minimal gating pathway are shown in the cyan and black circles, respectively. (**B)** The location of the biggest Grid_21_. (**C)** The structure of the biggest Grid_21_ with a 21-residue size to control the weakest N412-D512 H-bond at the pre-S1/VSLD interface. (**D)** The sequence of the biggest Grid_21_ to control the weakest N412-D512 H-bond highlighted in the blue box.

When the K169A mutation disrupted the highly-conserved K169-E751’ salt bridge swap at the ATP site (55–57), the R416-D519 H-bond at the pre-S1/VSLD interface, as well as the E418-R690 salt bridge at the pre-S1/TRP interface, were also broken at 4°C (Fig. 4A). Consequently, the Y448-Q529 and E501-H523 H-bonds were disconnected, leading to the replacement of the Y409-E702 H-bond with the T397-E704 one at the pre-S1/TRP interface, and the substitution of the T566-S576 H-bond with the D519-R567 one at the S4-S5 linker/VSLD interface.

Despite the intact E610-K649 H-bond as found in the biggest Grid_14_ of the closed state (Fig. 3), the channel remained open with the new biggest Grid_21_ at the VSLD/pre-S1/TRP interfaces due to the absence of a grid size bigger than 21 between the C- and N-termini beyond the PC-dependent minimal gating pathway from D396 to K705 (Figs. 4B–C & S1). This Grid_21_ had a size of 21 free residues via the shortest circular path from D512 to D519, R567, Y565, F441, W433, K432, E704, T397, N412 and back to D512 to regulate the least-stable D512-N412 H-bond at the pre-S1/VSLD interface (Fig. 4D). When this weakest H-bond was energetically equivalent to 1.0 basic H-bonds, the melting temperature threshold (T_m,th_) for heat unfolding was estimated to be around 22°C (Table 1). Therefore, the K169A mutant could still be open up to 22°C.

On the other hand, with the release of the PC lipid from the active vanilloid site promoting the formation of the stimulatory D519-R567 H-bond (49), the total noncovalent interactions and the total grid sizes along the PC-dependent minimal gating pathway from D396 to K705 decreased from 53 and 96 to 39 and 91, respectively. This resulted in an increase in systematic thermal instability (T_i_) from 1.81 to 2.33 (Table 1). In this regard, the K169A-induced open state was actually unstable even when the temperature was lowered to 4°C. Notably, the calculated cold sensitivity (Ω_10,cold_) was about 3.48, much lower than the experimental mean heat sensitivity (Q_10,heat_) of 22.2 (Table 1)(32). Therefore, heat activation of reduced TRPV3 was not initiated from the unfolding of the inter-subunit K169-E751’ salt bridge.

## Discussion

Thermosensitive TRPV1-4 channels have specific thresholds and high temperature sensitivity (Q_10_) for heat activation. While the parameters of TRPV1 and TRPV3 can be explained by a change in their thermoring structures along the PI/PC-dependent minimal gating pathway (48–49), they have not been examined for cold activation. According to the heat capacity mechanism that extensively applies to the protein, heat-responsive TRPV1-4 channels should also be activated by cold. When both cold and heat activations have the same starting point, their Q_10_ values should be comparable (52).

To test this hypothesis, this study first identified the thermal sensors or starters of rTRPV1-Δ(603–626) and hTRPV3 with matched thresholds for heat activations, and then investigated their cold sensitivities from the same and different starting points. The results showed that the cold-evoked open state was different from the heat-evoked one. Further, the cold and heat activations from the same and different staring points resulted in shared and unshared temperature coefficients or sensitivities. The calculated structural cold sensitivity (Ω_10, cold_) of rTRPV1-Δ(603–626) could mirror the functional heat sensitivity (Q_10,heat_) when both cold and heat activations started from the release of the PI lipid from the active vanilloid site to disrupt the nearby weakest bridge. In contrast, the calculated structural cold sensitivity (Ω_10, cold_) of hTRPV3 was much lower than the functional heat sensitivity (Q_10,heat_) when both cold and heat activations had distinct origins. Therefore, this study further confirmed that heat activation of TRPV1 and TRPV3 actually began with the heat unfolding of the weakest noncovalent link along the lipid-dependent minimal gating pathway from the pre-S1 domain to the TRP domain.

### Cold-evoked states are distinct from heat-evoked states

Removal of PI from the active vanilloid site of rTRPV1 with or without the pore turret at low and high temperatures resulted in different open states (Fig. 2A) (48). Firstly, the stimulatory R557-E570 H-bond at the S4-S5 linker/VSLD interface, necessary for the optimal heat-evoked activity temperature of 56°C, was absent in the cold-evoked open state. Secondly, the highly conserved noncovalent interactions D576-T685 and F580-L678 in the pore domain, crucial for the maximal heat-evoked activity temperature of 61°C, also disappeared in the cold-evoked open state. Thirdly, in addition to the common smallest Grid_0_ consisting of Y441, F516, Y554 and Y555, two more smallest thermorings appeared in the cold-evoked open state compared to the heat-evoked open state (Fig. 2A) (48). Therefore, compared with the higher melting threshold (T_m,th_) of 56°C and systematic thermal instability (T_i_) or flexibility of 1.65 for the heat-evoked open state, although the cold-evoked open state had the biggest Grid_9_ to control the least-stable F522-F543 bridge for the lower T_m,th_ of 41°C, its T_i_ was as low as 1.39 (Table 1) (48).

In contrast, the K169A mutation-induced open state of reduced hTRPV3 at 4°C still maintained the stimulatory D519-R567 H-bond at the S4-S5 linker/VSLD interface and the conserved D586-T680 and F590-L673 bridges in the pore domain (Fig. 4A) (49). The main difference was that the biggest Grid_21_ governed the weakest N412-D512 bridge for the melting threshold (T_m,th_) of 22°C of the cold-evoked reduced open state while the biggest Grid_9_ governed the weakest D586-T680 and F590-L673 bridges for the T_m,th_ of 61°C of the heat-evoked oxidized open state (Fig. 4A) (49). Therefore, the systematic thermal instability (Ti) or flexibility of the cold-evoked reduced open state was 2.33, higher than 1.20 of the heat-evoked oxidized state with the C612-C619 disulfide bond in the pore domain (Table 1) (49).

### The same starter results in a similar temperature coefficient

A recent study indicated that the least-stable Y401-R499 cation-π interaction in the biggest Grid_13_ along the PI-dependent minimal gating pathway from D388 to K710 is located at the pre-S1/VSLD interface and has a matched threshold (T_m,th_) of 43°C for the heat activation of the full-length rTRPV1 (48). This study further confirmed that the same noncovalent bridge at the pre-S1/VSLD interface was responsible for the matched threshold of 41°C for the heat activation of the rTRPV1 without the pore turret (603–626) (Table 1) (58). The heat-induced unfolding of this weakest Y401-R499 bridge has been confirmed by the cryo-EM structure of open rTRPV1 above 43°C (40). However, it is unclear if the heat activation of rTRPV1 primarily originated from the heat unfolding of this Y401-R499 bridge at the specific threshold. Since cold activation of rTRPV1-Δ(603–626) was directly initiated from the removal of PI from the active vanilloid site and the concurrent disruption of the least-stable Y401-R499 bridge along the same PI-dependent minimal gating pathway from D388 to K710, the comparable cold and heat sensitivity of 19 demonstrated that both cold and heat activations did start from the unfolding of the weakest Y401-R499 bridge at the specific temperature threshold (Table 1).

### Different starters have different temperature coefficients

In contrast, similar to the least-stable K614-N647 H-bond in the biggest Grid_11_ in the pore domain of reduced mTRPV3 (49), the weakest E610-K649 H-bond in the biggest Grid_14_ of reduced hTRPV3 also had a matching threshold of 52°C for heat activation (Table 1) (32). However, when the K169A mutation-induced cold activation at 4°C did not disrupt this weakest E610-K649 H-bond, the calculated structural thermosensitivity (Ω_10,cold_) of 3.48 was significantly lower than the measured functional heat sensitivity (Q_10, heat_) of 22.2 (Table 1) (32). This lower cold sensitivity was consistent with the slow channel dilation of the K169A mutant at the S2-S3 linker from 42°C to 25°C when compared with the stimulus of carvacrol or 2-aminoethoxydiphenylborane (2-APB) (59–60). In this regard, the initial heat activation of TRPV3 above 50°C was not initiated from the disruption of the swapping K169-E751’ salt bridge. Since the mirrored thermosensitivities of rTRPV1-Δ(603–626) have confirmed that heat activation primarily stemmed from the heat unfolding of the least-stable noncovalent bridge with the matched threshold, the initial heat activation of reduced TRPV3 may also originate from the heat-induced unfolding of the weakest E610-K649 H-bond along the PC-dependent minimal gating pathway from D396 to K705.

## Conclusions

Most heat-responsive TRPV1-4 channels have a higher activation threshold for detecting noxious heat stimuli. However, the primary modules for these specific thresholds have not been precisely defined due to a global cooperative conformational change across the entire protein during channel opening. This study demonstrates that an alternative cold activation pathway with a mirrored heat coefficient could be used to precisely define the primary modules for the specific thresholds if the same heat capacity mechanism for cold and heat unfolding transitions is involved. Therefore, even though different starting points trigger distinct cold or heat unfolding pathways allosterically, the symmetric temperature coefficient can be used to precisely define their common staring point as the primary module for the specific threshold.

## Materials and Methods

### Data mining resources

The cryo-EM 3D structures of the reduced hTRPV3 channel with MSP2N2 in the closed state at 4°C (PDB ID, 6UW4, model resolution = 3.20 Å) and the reduced hTRPV3-K169A channel with MSP2N2 in the open state at 4°C (PDB ID, 6UW6, model resolution = 3.70 Å) were analyzed to study K169A-induced cold sensitivity (57). As a control, the cryo-EM 3D structures of the reduced rTRPV1-Δ(604–626) channel with MSP2N2 in the closed state at 4°C (PDB ID, 5IRZ, model resolution = 3.28 Å) and in the open state at 25°C (PDB ID, 8U3L, model resolution = 3.70 Å) were studied to analyze cold sensitivity upon PI removal (53–54).

### Filtering noncovalent interactions

The stereo-selective and regio-selective inter-domain diagonal and intra-domain lateral noncovalent interactions along the PI or PC-dependent minimal gating pathway of rTRPV1-D(604–626) or hTRPV3/K169A from D388 to K710 or from D396 to K705 were primarily analyzed using UCSF Chimera. The interactions were then filtered by the same strict and consistent standard as previously used and confirmed (45–51). The examined noncovalent interactions included salt bridges, lone pair/CH/cation-π interactions and H-bonds between paired amino acid side chains. Specific cutoff distances and interaction angles for the different noncovalent interactions can be found in the online Supporting Information (Table S1-S4).

### Mapping topological grid network using graph theory

The same protocol that was previously described and validated was used to map the systematic fluidic grid-like noncovalent interaction mesh network in this study (45–51). In this network, a topological grid consisted of several nodes representing amino acids and linked nodes representing noncovalent interactions along the single polypeptide chain. Graph theory and the Floyd–Warshall algorithm (61) were used to determine a grid size as the shortest round path length to control the least-thermostable noncovalent interaction within the grid. The grid size also represented the minimal number of side chains of free or silent amino acids or atoms that did not participate in any noncovalent interaction in the given grid. Uncommon grid sizes were highlighted in black numbers on the network map alongside the Grid_s_ with an s-residue size. The total noncovalent interactions (*N*) and total grid sizes (S) along the PI or PC-dependent minimal gating pathway of rTRPV1-Δ(604–626) or hTRPV3/K159A from D388 to K710 or from D396 to K705 were calculated and displayed in black and cyan circles, respectively, next to the mesh network map for the calculation of systematic thermal instability (T_i_).

### Calculation of the melting temperature threshold (T_m,th_)

The melting temperature threshold $\left({\text{T}}_{\text{m,th}}\right)$ for the heat-induced unfolding of a specific grid was determined using an empirical equation and coefficients that were calibrated based on temperature-dependent structural data from various proteins, such as class I and II fructose aldolases, TRPV1, TRPV3, and TRPM8 (45–51):

(1) $${\text{T}}_{\text{m,th}}\left({}^{\circ}\text{C}\right)=34+\left(\text{n}-2\right)\text{×}10+\left(20-\text{s}\right)\times 2$$

where, n represents the total number of basic H-bonds (each approximately 1 kcal/mol) that are calculated to be roughly equivalent in stability to the least-stable noncovalent interaction controlled by the specific grid (62). The variable s denotes the grid size used to regulate the least-stable noncovalent interaction within the grid. Thus, the grid’s heat capacity will increase with a decrease in grid size or an increase in the number of equivalent basic H-bonds.

### Evaluation of the grid-based systemic thermal instability (T_i_)

The same empirical equation used in previous studies on temperature-dependent structures was utilized to calculate the systematic thermal instability $\left({\text{T}}_{\text{i}}\right)$ along the given polypeptide chain (45–51):

(2) $${\text{T}}_{\text{i}}=\text{S}/\text{N}$$

where, S and N are the total grid sizes and non-covalent interactions along a specific polypeptide chain. This calculation allows for evaluation of the protein’s compact conformational entropy or flexibility.

### Evaluation of the systematic temperature sensitivity

A gating transition of the thermosensitive TRPV1 or TRPV3 channel is always accompanied by a change in the energy density along the lipid-dependent minimal gating pathway (48–49). Accordingly, for enthalpy-driven activation of TRPV1 or TRPV3 from a closed state within 10°C as a result of the broken biggest grid, if the chemical potential of a grid is theoretically defined as the maximal potential for equivalent residues in the grid to form the tightest β-hairpin with the smallest loop via noncovalent interactions (62), the grid-based structural thermo-sensitivity (${\text{Ω}}_{10}$) of a single ion channel for cold activation could be defined and calculated using the following equations as examined previously (48–49).

(3) $${\text{Ω}}_{10}={\left[\left({\text{S}}_{\text{c}}-{\text{S}}_{\text{o}}\right)\text{E}/2\right]}^{\left(\text{Hc}/\text{Ho}\right)}={\left[\left({\text{S}}_{\text{c}}-{\text{S}}_{\text{o}}\right)\text{E}/2\right]}^{\left[\left(\text{ENc}/(\text{ENo}\right)\right]}={\left[\left({\text{S}}_{\text{c}}-{\text{S}}_{\text{o}}\right)\text{E}/2\right]}^{\left(\text{Nc}/\text{No}\right)}$$

where, along the same defined PI or PC-dependent minimal gating pathway of one subunit from D388 to K710 or from D396 to K705, N_c_ and N_o_ represent the total noncovalent interactions, H_c_ and H_o_ denote the total enthalpy included in them, and S_c_ and S_o_ indicate the total grid sizes in the closed and open states, respectively. The energy intensity of a noncovalent interaction is denoted by E and is typically 1 kcal/mol. Thus, Ω_10_ factually reflects a thermo-evoked change in the total chemical potential of grids upon a thermo-evoked change in the total enthalpy included in the noncovalent interactions apparently from a closed state to an open state along the same defined PI or PC-dependent minimal gating pathway of one subunit.

For a convenient comparison, the functional thermo-sensitivity (Q_10_) of a single ion channel for heat activation can be calculated using the following equation:

(4) $$Q_{10}={\left(X_{2}/X_{1}\right)}^{10/\left(T2-T1\right)}$$

where, X_1_ and X_2_ are the relative channel activity obtained at temperatures T1 and T2 (measured in Kelvin), respectively.

## Figures and Tables

**Table 1:** Comparison of local cold-induced thermoring structural changes of TRPV1 or TRPV3 along the PI or PC-dependent minimal gating pathway D388 to K710 or from D396 to K705. The comparative parameters are highlighted in bold.

PDB ID	5IRZ	8U3L	6UW4	6UW6
**Construct**	rTRPV1-Δ(604–626)	rTRPV1-Δ(604–626)	hTRPV3	hTRPV3-K169A
**Lipid PI/PC at the vanilloid site**	bound	free	present	free
**Redox state**	reduced	reduced	reduced	reduced
**Lipid environment**	MSP2N2	MSP2N2	MSP2N2	MSP2N2
**Sampling temperature, °C**	4	25	4	4
**Gating state**	Closed	Open	Closed	Open
**# of the biggest Grid_s_**	Grid_14_	Grid_9_	Grid_14′_	Grid_21_
**grid size (s)**	14	9	14	21
**# of energetically equivalent basic H-bonds (n) controlled by Grid_s_**	1.5	0.5	2.5	1.0
**Total non-covalent interactions (N)**	54	44	53	39
**Total grid sizes (S), a.a**	83	61	96	91
**Systemic thermal instability (T_i_)**	1.54	1.39	1.81	2.33
**Calculated T_m,th_, °C**	**41**	**41**	**51**	**22**
**Experimental T_m,th_, °C**	**41**	**< 41**	**50**	**< 25**
**Calculated Ω_10,cold_ at E = 1 kcal/mol**		19.1		3.48
**Experimental Q_10,heat_**		18.5		22.2
**Refs for Experimental T_m,th_, °C and Q_10_**	[58]	[58]	[32]	[32, 59]
**Figures and legends**				

## Data Availability

All data generated or analysed during this study are included in this published article and Supporting Information.

## ABBREVIATIONS

ARD: ankyrin repeat domain
cryo-EM: cryogenic electron microscopy
DSC: differential scanning calorimetry
Ω_10_: structural thermosensitivity
Q_10_: functional thermosensitivity
PI: phosphatidylinositol
PC: phosphatidylcholine
T_i_: systematic thermal instability
T_m,th_: melting temperature threshold
TRP: transient receptor potential
TRPV: TRP vanilloid
TRPVi: i=1, 2, 3, 4
TRPM2-5, 8: TRP melastatin 2-5, 8
TRPA1: TRP ankyrin 1
TRPC5: TRP canonical 5
rTRPV1: rat TRPV1
hTRPV3: human TRPV3
mTRPV3: mouse TRPV3
VSLD: voltage-sensor-like domain
2-APB: 2-aminoethoxydiphenylborane
WT: wild-type
